# A quasi-experimental study of fresh oxygen flow on patients’ oxygen reserve during mask-assisted ventilation under general anesthesia induction

**DOI:** 10.3389/fmed.2023.1261177

**Published:** 2023-09-13

**Authors:** Yubo Shi, Ying Jin, Jianli Song, Jingfeng Shi, Xiaoying Liu, Guoqing Zhao, Zhenbo Su

**Affiliations:** ^1^Department of Anesthesiology, China-Japan Union Hospital of Jilin University, Changchun, China; ^2^Department of Ultrasound, China-Japan Union Hospital of Jilin University, Changchun, China; ^3^Department of Anesthesiology, Zigong Fourth People’s Hospital, Zigong, China; ^4^Department of Anesthesia, Jiutai District Hospital of Traditional Chinese Medicine, Changchun, China

**Keywords:** pre-oxygenation, mask-assisted ventilation, fresh oxygen flow, safe apnea time, oxygen reserve

## Abstract

**Background:**

To compare the effect of different amounts of fresh oxygen flow on oxygen reserve in patients undergoing general anesthesia.

**Methods:**

Seventy-two patients were enrolled in this quasi-experimental study. Patients were randomly divided into experimental groups with a fresh oxygen flow of 1 L/min, 2 L/min, 4 L/min, and 8 L/min (denoted as G1, G2, G3, and G4, respectively) for 2 min of mask-assisted ventilation. Safe apnea time (SAT) was the primary endpoint; SAT was defined as the time from the cessation of ventilation to the time the patient’s pulse oxygen saturation (SpO_2_) decreased to 90%. Ventilation indicators such as end-tidal oxygen concentration (EtO_2_), end-tidal carbon dioxide partial pressure (EtCO_2_), SpO_2,_ and carbon dioxide (CO_2_) elimination amount, during mask-assisted ventilation, were the secondary endpoints.

**Results:**

The SAT of G1, G2, G3, and G4 were 305.1 ± 97.0 s, 315 ± 112.5 s, 381.3 ± 118.6 s, and 359 ± 104.4 s, respectively (*p* > 0.05). The EtO_2_ after 2 min of mask-assisted ventilation in groups G1, G2, G3, and G4 were 69.7 ± 8.8%, 75.2 ± 5.0%, 82.5 ± 3.3%, and 86.8 ± 1.5%, respectively (*p* < 0.05). Also, there was a moderate positive correlation between the fresh oxygen flow and EtO_2_ (correlation coefficient r = 0.52, 95% CI 0.31–0.67, *p* < 0.0001). The CO_2_ elimination in the G1 and G2 groups was greater than that in the G4 group (*p* < 0.05). There was no significant difference in other indicators among the groups (all *p* > 0.05).

**Conclusion:**

The amount of fresh oxygen flow during mask-assisted ventilation was positively correlated with EtO_2_. Also, even though there was no significant difference, the patients’ oxygen reserves increased with the increase in fresh oxygen flow.

## Introduction

Pre-oxygenation is a routine clinical anesthesia procedure given before anesthesia induction and tracheal intubation (1). Its purpose is to improve the oxygen reserve in the patient’s lungs and provide sufficient time for establishing an accurate artificial airway during anesthesia induction so as to avoid hypoxia (2–4). Previous studies have also shown that pre-oxygenation reduces hypoxemia during induction of anesthesia (5–7).

Pre-oxygenation during anesthesia induction is usually performed with pure oxygen. The commonly used clinical method is to pre-oxygenate the patient by tidal volume method under a fresh oxygen flow of 10 L/min for 3 min before induction of anesthesia (8). End-tidal oxygen concentration (EtO_2_) ≥90% or end-tidal nitrogen concentration (EtN_2_) <5% indicate maximal pre-oxygenation (1, 2, 9). When the optimal pre-oxygenation is achieved, the oxygen reserve in the patient’s lungs is significantly greater than the oxygen reserve in the patient’s lungs when breathing air, which can significantly extend the safe apnea time (SAT) of surgical patients to approximately 7 min (the SAT of normal people breathing air is about 1 min) (7, 10). Due to the unpredictable occurrence of some difficult airways, pre-oxygenation is strongly recommended before induction of anesthesia in patients undergoing general anesthesia surgery, as it extends the operating time for establishing an artificial airway (3, 4, 7, 10, 11).

Hypoxaemia remains the most common cause of death, especially during induction of anesthesia, as reported by the American Society of Anesthesiologists (ASA) Closed Claims analysis (12). Many studies have demonstrated that pre-oxygenation can increase the oxygen reserve stored in the functional residual capacity and delay the onset of hypoxemia in case of unanticipated difficult airway management (3, 4, 7, 11). However, the prevalence of inadequate preoxygenation has been reported to be as high as 56% during the induction of anesthesia (13). This condition is mainly caused by air leakage and risk factors associated with difficult mask ventilation (13–15). Currently, there is no clear standard for the amount of fresh oxygen flow during mask-assisted ventilation. Thus, in this study, we compared the effect of different amounts of fresh oxygen flow on oxygen reserve in patients undergoing general anesthesia surgery so as to provide a reliable reference value for the fresh oxygen flow during mask-assisted ventilation in clinical anesthesia.

## Materials and methods

### Study design and patients

This quasi-experiment study included patients undergoing elective surgery with general anesthesia at the China-Japan Union Hospital of Jilin University, between October 2021 and January 2022. The study was approved by the Ethics Committee of China-Japan Union Hospital of Jilin University (No: 2021092711). Written informed consents were obtained from all subjects.

A total of 72 adult patients (age 18–60 years; BMI 18–30 kg/m^2^) with American Society of Anaesthesiologists (ASA) physical Status I to II scheduled for elective surgery under general anaesthesia were included in this study. Patients with room air saturation of <97%, anticipated difficult mask ventilation and anticipated difficult tracheal intubation, chronic pulmonary diseases, ischemic heart or brain diseases, raised intracranial pressure, risk of reflux aspiration, facial deformities, and those who were pregnant were excluded.

### Randomization

Random number sequences were generated by computer using *SPSS ver. 23.0* (*IBM, Armonk, New York, United States*) software. Patients were randomly assigned to 4 groups (18 people per group): groups G1 (fresh oxygen flow = 1 L/min), G2 (fresh oxygen flow = 2 L/min), G3 (fresh oxygen flow = 4 L/min), and G4 group (fresh oxygen flow = 8 L/min). Allocation concealment was done in a blind manner, using sequentially numbered opaque sealed envelopes.

### Intervention

All patients fasted for 8 h before surgery. After arriving in the operating room, all patients rested flat in a supine position on the operating table and were monitored with an electrocardiogram (ECG), non-invasive blood pressure (NIBP), bispectral index (BIS), and pulse oximetry. At the same time, we connected a sensor (Mindray RM, Shenzhen, Guangdong, China) at the Y-shaped interface of the breathing circuit to collect the carbon dioxide partial pressure, gas flow rate, pressure, and volume data in the breathing circuit, which were subsequently exported through the computer at a frequency of 50 Hz for analysis and use. Before anesthesia induction, all patients received pre-oxygenation by tidal volume method for 3 min at an oxygen flow of 10 L/min, and the mask was kept as tight as possible. General anesthesia was induced with lidocaine 1 mg/kg, sufentanyl 0.4 μg/kg, propofol 2 mg/kg and cisatracurium 0.2 mg/kg. When the carbon dioxide waveform became flat, mask-assisted ventilation was started in pressure control mode for 2 min (I:E = 1:2, maximum inspiratory pressure 15 cmH_2_O, respiratory rate 15 breaths/min). During mask-assisted ventilation, the fresh oxygen flow was set to 1 L/min, 2 L/min, 4 L/min, and 8 L/min according to the randomization. All patients were used jaw thrust method to ensure airway patency and the carbon dioxide waveform was monitored to ensure the best fit between the mask and the face of each patient. All patients were intubated with a video laryngoscope, and the cuff was inflated after the tracheal tube passed through the glottis under direct vision. After tracheal intubation, ventilation was temporarily disconnected, and mechanical ventilation was not started until SpO_2_ dropped to 90% or SAT reached 10 min (6). If SpO_2_ fell below 98% after mask-assisted ventilation or SpO_2_ dropped below 90% during intubation, or difficult endotracheal intubation occurred, the operation was considered as not successful.

When the patient’s SpO_2_ dropped to 90% or SAT reached 10 min, volume-controlled mechanical ventilation was initiated. Tidal volume was set to 8 mL/kg based on predicted body weight, the fresh oxygen flow was 10 L/min, the respiratory rate was 15 breaths/min, and the I:E was 1:2. The entire experiment ended when the patient’s pulse oximetry reached the highest level. According to the patient’s condition, respiratory rate, inhaled oxygen concentration, and fresh oxygen flow were re-adjusted if necessary.

### Outcomes and measurement

Baseline characteristics included age, gender, height, weight, BMI, IBW, HR, SpO_2,_ and hemoglobin values. SAT was the primary endpoint; ventilation indicators such as EtO_2_, EtCO_2_, SpO_2,_ CO_2_ elimination amount, etc., during mask-assisted ventilation were the secondary endpoints. The SAT was defined as the time spent from the end of mask-assisted ventilation until the SpO_2_ dropped to 90% or lasting for a maximum of 10 min to ensure patient safety (6); the time from the end of mask-assisted ventilation to the time when SpO_2_ dropped to 99%, 97%, 95%, 93%, and 90% were recorded. When the patient’s SpO_2_ was >90% after the SAT reached 10 min, it was recorded as 600 s; SpO_2_, EtO_2_ and EtCO_2_ of the patient after 3 min of pre-oxygenation were recorded; SpO_2_, EtO_2_, EtCO_2_, and HR of the patient after 2 min of mask-assisted ventilation were recorded; time during which the patient’s SpO_2_ recovered to the level after pre-oxygenation since the start of mechanical ventilation, i.e., the re-oxygenation time was recorded; EtCO_2_ and HR when the patient started mechanical ventilation were recorded; the patient’s lowest SpO_2_ throughout the experiment was recorded; CO_2_ elimination amount during the patient’s 2-min mask-assisted ventilation [the amount of CO_2_ elimination (mL) = ∑[(Flow/60) × (CO_2_ partial pressure/760) × 0.02]; the flow was calculated as the expiratory gas flow rate in mL/min; data export frequency was 50 Hz].

### Statistical analysis

A previous study that used pure oxygen pre-oxygenation in general anesthesia surgery noted the mean SAT of 411 ± 84 (95% CI: 239–528) seconds (7). Based on these findings, the sample size needed to show a 15% change in the SAT with 80% power and an α-error of 0.05 was 15 patients in each group. Considering some dropouts, we decided to include 18 patients in each group.

Statistical analysis was performed using *SPSS ver. 23.0* (*IBM, Armonk, New York, United States*), and the drawing software was *Graphpad Prism version 8.0.2 (GraphPad Software, La Jolla, California, United States)*. If the continuous data conformed to the normal distribution, they were expressed as the mean ± standard deviation ( $\bar{x}$ *±sd*); the comparison between groups was performed by variance analysis, and the multi-sample mean comparison was performed by the *Student–Newman–Keuls* test method. If the continuous data conformed to the skewed distribution, they were expressed as the median (interquartile range) [*Median (Q1, Q3)*], and the multi-sample nonparametric rank-sum test (*Kruskal Wallis*) was used for comparison between groups. Categorical data were compared using the chi-square test. *p* < 0.05 was considered to be statistically significant.

## Results

**Figure 1:**
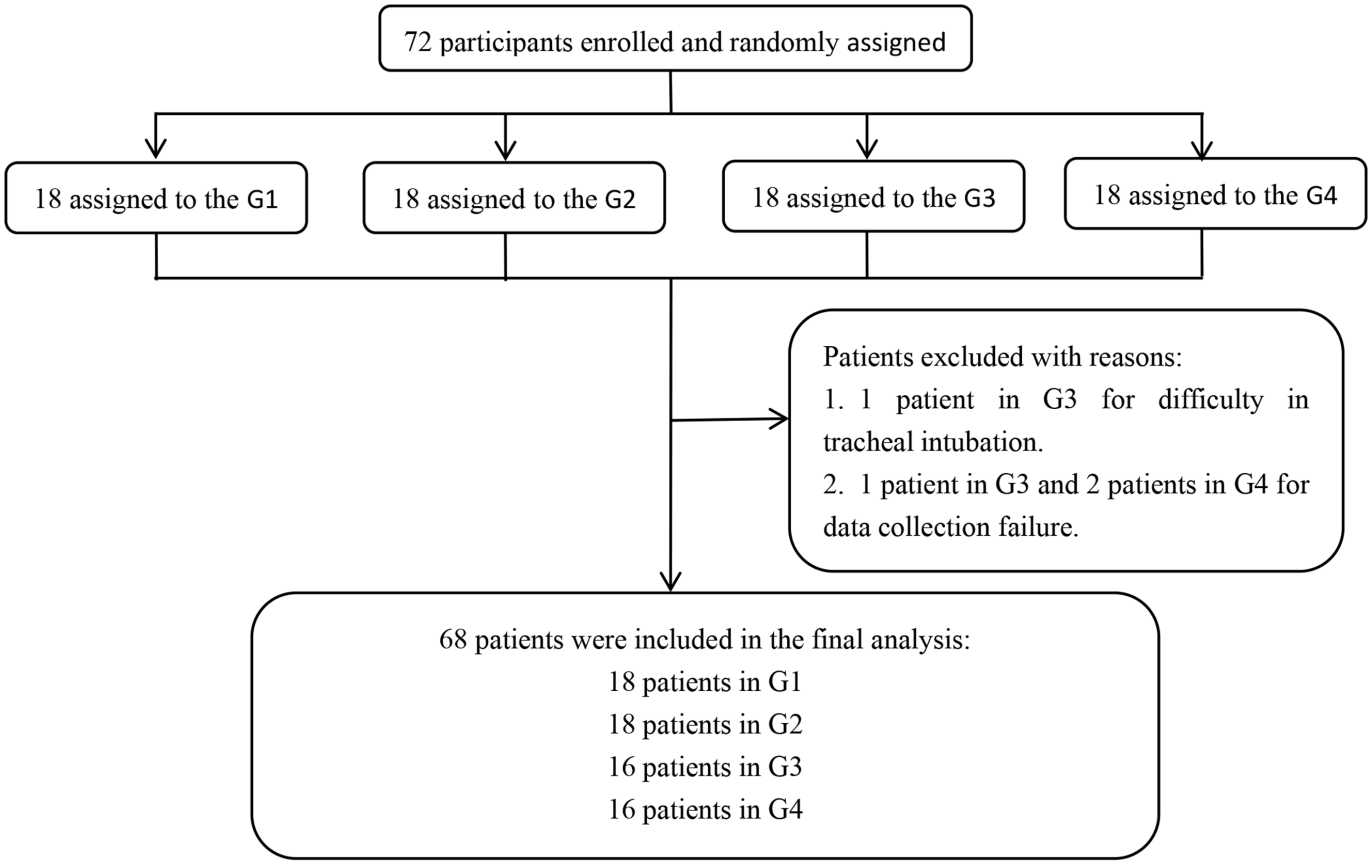
Experimental flow chart. G1, G2, G3, and G4 represent the fresh oxygen flow of 1 L/min, 2 L/min, 4 L/min, and 8 L/min, respectively.

A total of 72 patients were initially evaluated. Two patients in the G3 were excluded due to difficult endotracheal intubation and failed data collection, respectively, and 2 patients in the G4 were excluded due to failed data collection. Finally, 68 patients completed the experiment, 18 patients in G1, 18 patients in G2, 16 patients in G3, and 16 patients in G4. The experimental flow chart is shown in Figure 1. Baseline characteristics of study patients were not significantly different among the 4 groups (all *p* > 0.05, Table 1).

**Table 1:** Baseline characteristics of study patients.

	G1 (*N* = 18)	G2 (*N* = 18)	G3 (*N* = 16)	G4 (*N* = 16)	*p* value
Sex (male/female)	10/8	9/9	7/9	5/11	0.540
Age (year)	40.6 ± 11.9	44.8 ± 9.4	40.9 ± 11.0	41.6 ± 10.6	0.666
Height (cm)	166.3 ± 7.1	168.4 ± 8.0	164.9 ± 6.4	164.5 ± 7.3	0.405
Weight (kg)	65.3 ± 11.1	68.9 ± 10.9	63.9 ± 7.9	68.0 ± 8.4	0.451
IBW (kg)	60.5 ± 8.2	62.3 ± 9.4	58.6 ± 7.7	57.9 ± 8.3	0.438
BMI (kg m^−2^)	23.8 ± 2.6	24.2 ± 2.9	23.5 ± 2.3	25.0 ± 1.6	0.263
Hb (g/dL)	141.8 ± 12.8	141.8 ± 17.0	136.7 ± 29.0	139.6 ± 15.0	0.867

Data presented as mean (±SD) or numbers when applicable. G1, G2, G3, and G4 represent the fresh oxygen flow of 1 L/min, 2 L/min, 4 L/min, and 8 L/min, respectively. The calculation formula is: Male (kg) = 50 + 0.91 × [Height (cm)−152.4], Female (kg) = 45.5 + 0.91 × [Height (cm)−152.4]. IBW, ideal body weight; BMI, body mass index; Hb, hemoglobin; FGF, fresh gas flow.

### Safe apnea time (SAT)

**Figure 2:**
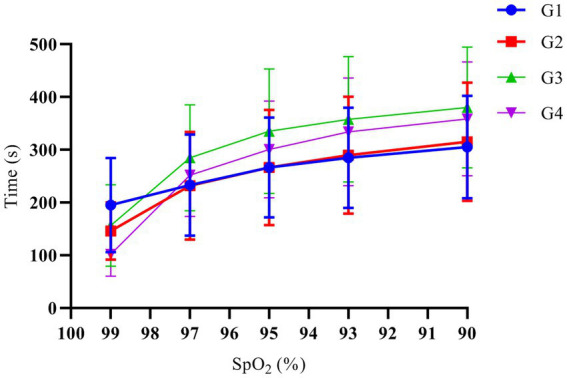
The variation trend of SpO_2_ over time. G1, G2, G3, and G4 represent the fresh oxygen flow of 1 L/min, 2 L/min, 4 L/min and 8 L/min, respectively. There was no significant difference in the time when the SpO_2_ decreased to 99%, 97%, 95%, 93%, and 90% among the groups (*p* > 0.05).

Among the 68 patients, 2 patients in G3 had an SAT of 600 s, while for all remaining patients, it was <600 s. G1, G2, G3, and G4 patients had SAT of 305.1 ± 97.0 s, 315 ± 112.5 s, 381.3 ± 118.6 s, and 359 ± 104.4 s, respectively; yet, the multi-sample mean comparison showed no statistical difference among the 4 groups (*p* = 0.139). In addition, the multi-sample mean comparison showed no statistical difference in the time when the SpO_2_ decreased to 99%, 97%, 95%, and 93% after mask-assisted ventilation (*p* > 0.05). The variation trend of SpO_2_ over time in each group of patients is shown in Figure 2.

### End-tidal oxygen concentration (EtO_2_)

**Figure 3:**
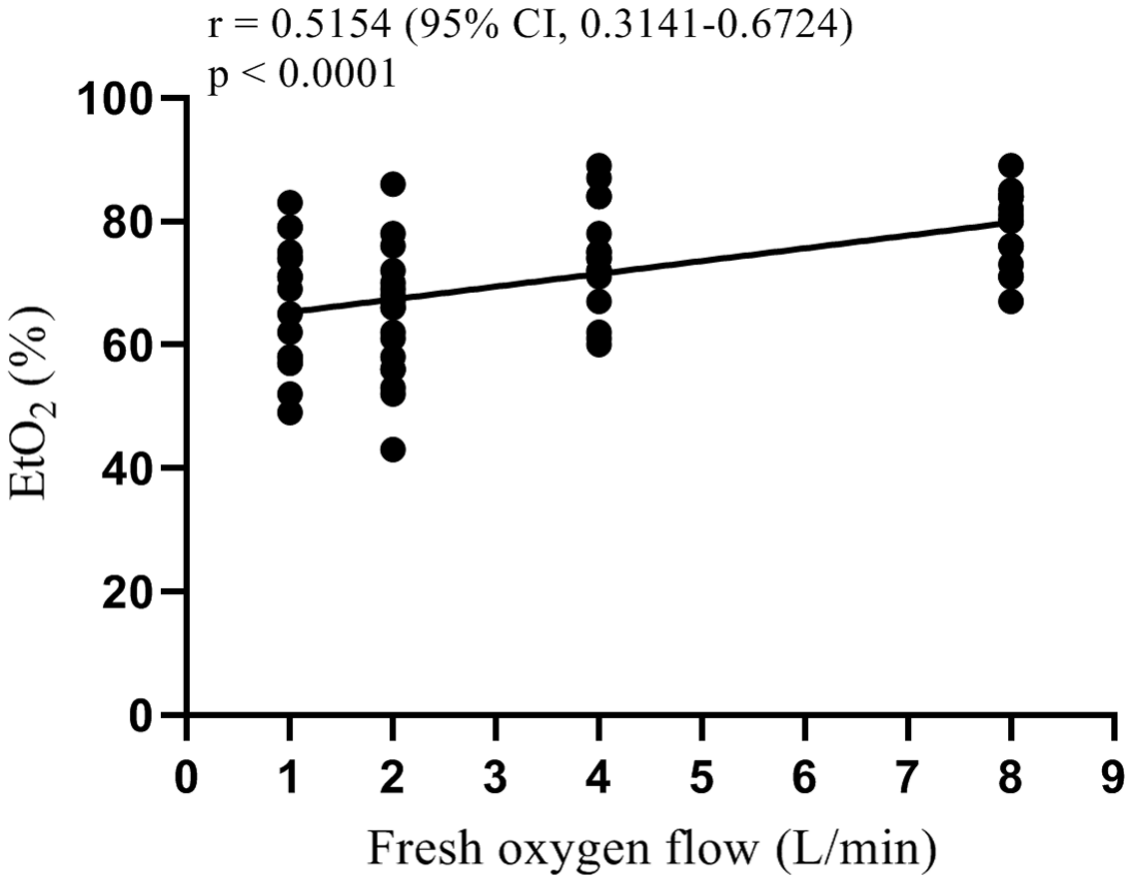
Scatter plot and correlation analysis of fresh oxygen flow and EtO_2_.

One-way analysis of variance showed that the end-tidal oxygen concentration of patients in each group was different after 3 min of pre-oxygenation and 2 min after mask-assisted ventilation (all *p* < 0.001, Table 2). The multi-sample mean comparison showed that G1, G2, G3, and G4 had statistical differences in EtO_2_ after 2 min of mask-assisted ventilation (*p* < 0.05). Further, Pearson correlation analysis showed that the fresh oxygen flow had a moderately strong correlation with EtO_2_ during mask-assisted ventilation (correlation coefficient *r* = 0.52, 95% CI 0.31–0.67, *p* < 0.001, Figure 3).

**Table 2:** Comparison of EtO_2_ at different time points in each group.

	G1 (*N* = 18)	G2 (*N* = 18)	G3 (*N* = 16)	G4 (*N* = 16)	*p* value
EtO_2_ (%) after 3 min of pre-oxygenation*	66.7 ± 9.9	64.4 ± 10.5	74.1 ± 8.9	79.2 ± 6.2	<0.001
After 2 min of MAV*	69.7 ± 8.8	75.2 ± 5.0	82.5 ± 3.3	86.8 ± 1.5	<0.001
EtCO_2_ (mmHg) after 3 min of pre-oxygenation	31.4 ± 5.2	32.3 ± 5.3	28.8 ± 5.3	29.1 ± 5.3	0.115
After 2 min of MAV	31.3 ± 2.4	30.8 ± 2.7	28.9 ± 4.4	29.4 ± 4.0	0.148
When starting MV	47.1 ± 6.8	46.6 ± 4.5	48.6 ± 6.6	46.9 ± 3.8	0.760
SpO_2_ (%) at baseline	97.0 (96.0, 98.0)	96.5 (95.8, 98.0)	96.5 (96.0, 98.0)	96.0 (95.3, 97.0)	0.363
After 3 min of pre-oxygenation	99.5 (99.0, 100.0)	100.0 (99.0, 100.0)	99.5 (99.0, 100.0)	100.0 (99.0, 100.0)	0.969
After 2 min of MAV	100.0 (99.0, 100.0)	100.0 (99.0, 100.0)	99.0 (99.0, 100.0)	99.0 (99.0, 100.0)	0.427
The lowest SpO_2_	86.0 (85.8, 88.0)	87.5 (86.0, 88.0)	87.5 (87.0, 88.0)	88.0 (86.0, 88.0)	0.105
Reoxygenation time (s)	44.0 (36.8, 48.0)	41.0 (33.5, 48.0)	45.5 (29.3, 57.8)	34.5 (30.0, 42.0)	0.947
HR (times/min) at baseline	79.5 (67.8, 91.3)	70.0 (67.5, 80.0)	78.5 (64.0, 91.8)	81.5 (75.3, 101.3)	0.058
After 2 min of MAV	68.5 (58.8, 81.0)	62.0 (55.3, 69.5)	66.5 (58.5, 73.8)	69.0 (64.5, 77.0)	0.095
When SpO_2_ is 90%	70.0 (62.8, 85.8)	65.5 (61.28, 76.5)	68.0 (63.3, 73.5)	72.0 (65.3, 79.5)	0.394

Data are presented as mean (±SD) or median (interquartile range). G1, G2, G3, and G4 represent the fresh oxygen flow of 1 L/min, 2 L/min, 4 L/min, and 8 L/min, respectively. FGF, fresh gas flow; MAV, mask-assisted ventilation; MV, mechanical ventilation; * indicates that the statistical results of one-way ANOVA among groups are *p* < 0.05.

### CO_2_ elimination amount

**Figure 4:**
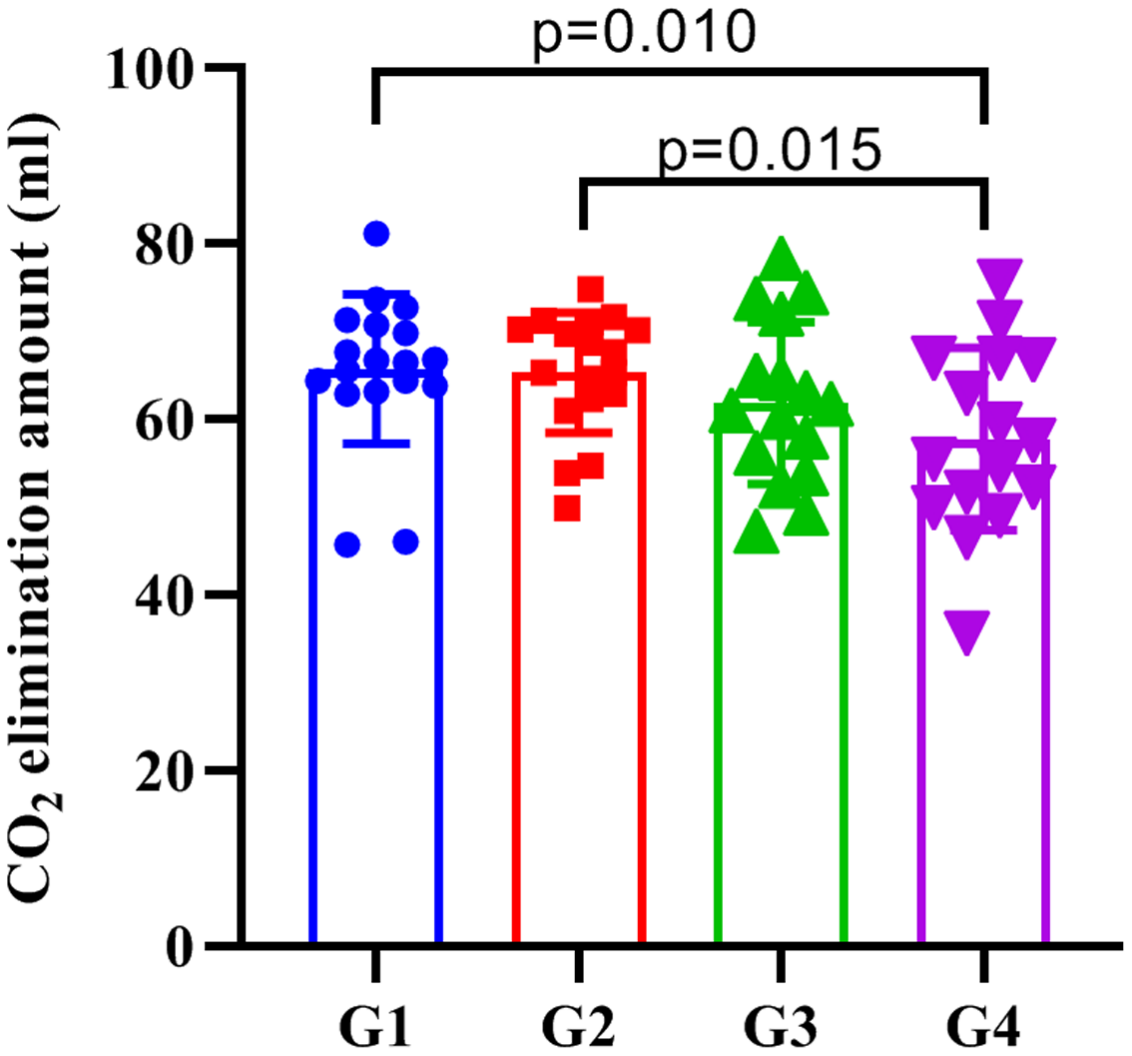
The amount of CO_2_ elimination in each group. G1, G2, G3, and G4 represent the fresh oxygen flow of 1 L/min, 2 L/min, 4 L/min, and 8 L/min, respectively. The CO_2_ elimination of the patients in the G1 and G2 was greater than G4 (*p* = 0.010 and *p* = 0.015), while G3 did not significantly differ from the other three groups (*p* > 0.05).

One-way analysis of variance showed that the CO_2_ elimination amount of patients in each group were statistically different (*p* = 0.038). The multi-sample mean comparison of Student–Newman–Keuls found that the CO_2_ elimination amount of the patients in the G1 and G2 was greater than G4 (*p* = 0.010 and *p* = 0.015), while G3 had no significant difference with other groups (*p* > 0.05, Figure 4).

### End-tidal carbon dioxide partial pressure (EtCO_2_)

There was no significant difference in EtCO_2_ between groups of patients after 3 min of pre-oxygenation, 2 min after mask-assisted ventilation, and when mechanical ventilation was started (all *p* > 0.05, Table 2).

### Pulse oxygen saturation (SpO_2_)

There was no significant difference in the SpO_2_ at baseline, 3 min after pre-oxygenation, 2 min after mask-assisted ventilation, and the lowest SpO_2_ after stopping ventilation (all *p* > 0.05, Table 2).

### Re-oxygenation time

Mechanical ventilation was started when the SpO_2_ of patients in each group dropped to 90%, and the nonparametric rank-sum test showed no significant difference in re-oxygenation time among the groups (*p* > 0.05, Table 2).

### Heart rate (HR)

There was no significant difference in the HR of patients in each group at baseline, after 2 min of mask-assisted ventilation, and when the SpO_2_ was 90% (all *p* > 0.05, Table 2).

## Discussion

The key findings of this study are: (1) no significant difference was observed for SAT among patients during mask-assisted ventilation when the fresh oxygen flow varied from 1 to 8 L/min, but there was a significant trend toward prolongation; (2) during mask-assisted ventilation, the EtO_2_ showed a moderately strong correlation with the fresh oxygen flow. These findings indicate that the amount of fresh oxygen flow during mask-assisted ventilation affects the patient’s oxygen reserve.

SAT is defined as the interval between the onset of apnea and the time SpO_2_ reaches a life-threatening value and is usually defined in clinical studies as SpO_2_ ≤ 90%, which can reflect the oxygen reserve in the patient’s body (4, 6, 7). According to the sigmoid shape of the oxyhemoglobin dissociation curve, SAT is defined as a decrease in SpO_2_ to 90% that is not harmful to most healthy patients. Altitude experiments have indicated that patients can tolerate periods of relative hypoxia lasting hours or days (SpO_2_ = 80% or less) (16–18). In this study, we evaluated the effect on the oxygen reserve of patients by adjusting the fresh oxygen flow during anesthesia induction in patients with adequate pre-oxygenation, and found no significant difference in SAT when the fresh oxygen flow was 1–8 L/min. However, with the increase of fresh oxygen flow, we observed a clear prolongation trend of SAT. The SAT of the G4 group was shorter than that of the G3 group, which may be due to the higher BMI in the G4 group. Yet, a larger sample size study is needed to confirm these data.

EtO_2_, a simple and effective endpoint for pre-oxygenation, showed a moderately strong positive correlation with fresh oxygen flow in this study. This finding is partially consistent with previous studies reporting on fresh oxygen flow during pre-oxygenation (19, 20). Nimmagadda et al. performed pre-oxygenation with fresh oxygen flow groups of 5, 7, and 10 L/min and showed that the EtO_2_ increased rapidly between 0.5 and 2 min, reaching a plateau at 2.5 min. In addition, pairwise survival analysis showed no difference in patient’s time to the target EtO_2_ = 90% among the three washing curves (19). Moreover, Russell et al. applied a fresh oxygen flow of 5, 10, and 15 L/min during pre-oxygenation and found the best pre-oxygenation effect when the flow was greater than 10 L/min (20). Similar conclusions were drawn in this study, where we observed a positive correlation with EtO_2_ in patients with increasing fresh oxygen flow, but we did not observe a plateau during the 2 min of mask-assisted ventilation period. However, the EtO_2_ value alone could not accurately predict the SAT of the patient as it is related to the patient’s functional residual capacity, oxygen consumption, and the amount of oxygen required to maintain SpO_2_ = 90% (4, 18).

In this study, we also compared the CO_2_ elimination amount and EtCO_2_ at different time points in each group, which can reflect the ventilation efficiency of patients (16). Previous studies have shown that the amount of CO_2_ elimination is related to the amount of blood flow in pulmonary capillaries, the gradient of the partial pressure between alveoli and capillaries, the blood/alveoli membrane characteristics and the properties of the ventilation cycle, and its main influencing factors include abnormal lung function, gender and weight of patients (21, 22). This study found that the amount of CO_2_ elimination in the G1 and G2 groups was higher than that in the G4 group, while there was no significant difference between the G3 and G4 groups. This may be due to the low flow of fresh oxygen in patients in groups G1 and G2, resulting in higher CO2 partial pressure in the breathing circuit. At the same time, low FGF will also increase the partial pressure of non-oxygen gases, thereby reducing the FiO2 and affecting the patient’s oxygen reserve. Therefore, we recommend fresh oxygen flow ≥4 L/min during mask-assisted ventilation to avoid an increase in non-oxygen gas partial pressure in the breathing circuit.

This study has several limitations. First, the primary aim SAT of this study was not significantly different among the groups, which may be due to the small sample size or individual differences. Second, air leakage during the pre-oxygenation process resulted in differences in the EtO_2_ among groups after 3 min of pre-oxygenation, which had a certain impact on the subsequent experimental results. Third, blood gas analysis indicators, hemodynamic indicators, and assessment of the occurrence of atelectasis were not included in the experimental observation indicators, which are of great significance to the experimental integrity and patient safety.

To sum up, this is the first randomized controlled trial that compared the effect of fresh oxygen flow on patient oxygen reserve during mask-assisted ventilation in adequately pre-oxygenated patients. Our results indicated that the amount of fresh oxygen flow during mask-assisted ventilation was positively correlated with EtO_2_. At the same time, although there was no significant difference in the oxygen reserve of the patients in each group, the patients’ oxygen reserves tended to increase with the increase of fresh oxygen flow. Thus, we recommend fresh oxygen flow ≥4 L/min during mask-assisted ventilation to avoid an increase in non-oxygen gas partial pressure in the breathing circuit.

## Data availability statement

The original contributions presented in the study are included in the article/supplementary material, further inquiries can be directed to the corresponding author.

## Ethics statement

The studies involving humans were approved by the study was approved by the Ethics Committee of China-Japan Union Hospital of Jilin University (No: 2021092711). The studies were conducted in accordance with the local legislation and institutional requirements. The participants provided their written informed consent to participate in this study.

## Author contributions

YS: conceptualization, data curation, writing – original draft, writing – review and editing. YJ: conceptualization, data curation, writing – original draft, writing – review and editing. JSO: data curation, formal analysis, writing – original draft, writing – review and editing. JSH: data curation, formal analysis, writing – original draft, writing – review and editing. XL: data curation, formal analysis, writing – original draft, writing – review and editing. GZ: data curation, formal analysis, writing – original draft, writing – review and editing. ZS: conceptualization, writing – original draft, writing – review and editing.

## Funding

This study was supported by the Key R&D Project of Jilin Province Science and Technology Development Program (no.202020404165YY).

## Conflict of interest

The authors declare that the research was conducted in the absence of any commercial or financial relationships that could be construed as a potential conflict of interest.

## Publisher’s note

All claims expressed in this article are solely those of the authors and do not necessarily represent those of their affiliated organizations, or those of the publisher, the editors and the reviewers. Any product that may be evaluated in this article, or claim that may be made by its manufacturer, is not guaranteed or endorsed by the publisher.
